# Intraocular pressure response affected by changing of sitting and supine positions

**DOI:** 10.1111/aos.14267

**Published:** 2019-10-10

**Authors:** Eliška Najmanová, František Pluháček, Markéta Haklová

**Affiliations:** ^1^ Faculty of Science Department of Optics Palacký University Olomouc Olomouc Czech Republic

**Keywords:** baseline, body position, intraocular pressure, sitting, supine, time course

## Abstract

**Purpose:**

To assess the intraocular pressure (IOP) time response to change in body position from sitting to supine and from supine to sitting immediately and during rest in each position.

**Methods:**

Forty‐four visually healthy volunteers were recruited for the study. The experiment consisted of the initial sitting position (baseline state), the subsequent lying period and the final sitting period. Both periods were 30 min long. The IOP was measured in the baseline state, immediately after each position change and then in minutes 5, 15, 25 and 30 during each period. The Icare Pro^®^ rebound tonometer was used.

**Results:**

The mean IOP increased after each position change (2.6 ± 2.4 mmHg after lying down and 2.1 ± 3.1 mmHg after sitting up) and then gradually decreased with time. The mean IOP was 1.41 ± 2.4 mmHg higher in the lying period than in the sitting period; the mean difference was smaller for the lower baseline (0.9 ± 2.2 mmHg) than the higher baseline (1.9 ± 2.5 mmHg). The mean IOP in the final sitting was significantly lower (2.5 ± 1.9 mmHg) than in the initial sitting position. The effect of sex was insignificant.

**Conclusions:**

There was an immediate increase in IOP as a response to both changes in the body position and the subsequent gradual decrease with time. The IOP difference between lying and sitting position was depended on baseline.

## Purpose

Intraocular pressure (IOP) is one of the major risk factors of glaucoma, and its monitoring is an important part of glaucoma screening and diagnostics (Allingham et al. 2010). There are many factors influencing IOP value, for example physical activity (Najmanova et al. 2016, 2018; Vera et al. 2018), hypoxia (Cymerman et al. 2000; Ersanli et al. 2006; Pavlidis et al. 2006; Karadaq et al. 2008; Najmanová et al. 2019), drinking of water (Read & Collins 2010; Salcedo et al. 2018; Susanna et al. 2018), etc. Intraocular pressure (IOP) is significantly affected by body position as well (e.g. Jorge et al. 2010; Fang et al. 2018; Kiuchi et al. 2010; Lam et al. 2013; Lee et al. 2012; Lindén et al. 2018; Malihi & Sit 2012; Meurs et al. 2018; for a review of older studies see Prata et al. 2010). The most studies refer to the higher IOP values in the supine compared to the sitting or upright position (e.g. Galin et al. 1963; Linder et al. 1988; Jorge et al. 2010; Kiuchi et al. 2010; Lee et al. 2012; Malihi & Sit 2012; Lam et al. 2013; Fang et al. 2018; Lindén et al. 2018; Meurs et al. 2018). Such an increase in IOP as well as its quick changes can be a risk factor for development and progression of glaucoma (e.g. Krist et al. 2001; Goldberg 2003; Hasegawa et al. 2006).

As IOP is usually measured in the sitting position due to technical facilities, there is less knowledge about the details of IOP dynamics after the position change. Moreover, many patients are transported in the supine position. As the common measurement position is sitting, the interpretation of measured values can be complicated by short‐time IOP changes associated with the body reposition before measurement.

Thus, regarding the possible increase of the risk of glaucoma damage as well as the proper IOP value determination, it is important to know the time response of IOP related to the position change. As glaucoma patients suffer from higher IOP, the effect of initial IOP is important as well. The purpose of this study was to assess the IOP time response to change in body position from sitting to supine and from supine to sitting immediately and during 30 min of rest in each position in healthy subjects. The influences of IOP baseline and sex were studied as well.

## Subjects and methods

Forty‐four visually healthy volunteers (12 men and 38 women) between the ages of 20 and 48 with a mean age of 24 and a standard deviation of 5 years were recruited for the study. Subjects were not allowed to have any evidence either of glaucomatous optic neuropathy or ocular hypertension. The subjects were also required to be free of ocular diseases which could affect IOP or its measurement such as keratoconus or high spherical defect (equal or greater than 3 dioptre) and corneal astigmatism (equal or greater than 2.5 dioptre). The subjects were not allowed to wear contact lenses for 12 hr prior to measurement. The subjects were asked to avoid all caffeine consumption or substances, and also avoid physical activity, which might affect IOP a day before the measurement. The subjects could drink only an essential amount of liquid (up to 2 decilitres) in the morning before and no liquids immediately an hour before the first measurement. The research followed the principles of the Declaration of Helsinki. Informed consent was obtained before any measurements were carried out on the subjects.

The experiment consisted of three main parts – the initial sitting position as a baseline state, the lying period and the final sitting period. The initial IOP (*IOP*_B_) was measured in the initial sitting position after 10 min of rest and was considered the baseline. Each subject then lay down and remained 30 min in a supine position. The subjects consequently sat up and remained 30 min in a sitting position. The subjects stayed in the same place (on the laboratory lounger), that is, did not move between the position changes. The subjects always changed the position themselves; each change was made within 10 seconds. The IOP was measured immediately after laying down and then in minutes 5, 15, 25 and 30 in the supine position, and immediately after sitting up and in minutes 5, 15, 25 and 30 in the sitting position. The delay between the last measurement in the previous position and the first measurement after the change was up to 20 seconds. All the measurements were finished in the morning time, which seems to be optimal for IOP measurement in order to eliminate the effect of circadian oscillation of IOP (Duke‐Elder 1952; Wilensky et al. 1993).

In the sitting position, the upper half of the body including the head was in the straight position; arms loosely along the body and the legs at a right angle. The subjects were lying down on their backs with arms and legs drawn out free loosely along the body. The head was always in the body axis. The eyes in both the supine and sitting position were open (expect for normal blinking), without dioptric correction and subjects should look ahead.

Intraocular pressure (IOP) was measured using Icare Pro^®^ rebound tonometer (Vantaa, Finland; http://www.icaretonometer.com), which is suitable for measurement in the sitting and supine position as well. The tonometer averaged six automatically measured consecutive readings and provided their mean IOP out, which was used in the analysis. The coefficient of the variation of the output (the automatically averaged IOP value) declared by the manufacturer is less than 8% in accordance with publication (Schweier et al. 2013). The variation is slightly higher in the reclining position (6.9%) compared with the sitting position (5.2%) (Schweier et al. 2013). Only the right eye of each participant was measured. A new probe was used for each measurement. All IOP measurements were administrated by one trained professional.

The time course of IOP values was analysed by one‐factor (time) repeated‐measures analysis of variance (anova). The effects of IOP baseline, sex, position and time on IOP changes from the baseline were analysed by four‐factor (baseline and sex as between factors, position and time as within factors) repeated‐measures anova. When necessary, the levels of statistical significance included a Huynh–Feldt correction for departures from sphericity. The post hoc pairwise comparisons were realized using a Tukey honest significant difference (HSD) test. For anova purposes, the subjects were divided by IOP baseline into lower (*IOP*_B_ < 17.8 mmHg) and higher (*IOP*_B_ ≥ 17.8 mmHg) groups based on the median value 17.8 mmHg. The potential relationships between IOP and other parameters were also studied by the Pearson correlation coefficient *r*. The significance level was set at 0.05. Data are presented as mean ± standard deviations. Statistical analyses were performed using STATISTICA 13.0 (StatSoft, Tulsa, OK, USA).

## Results

**Figure 1:**
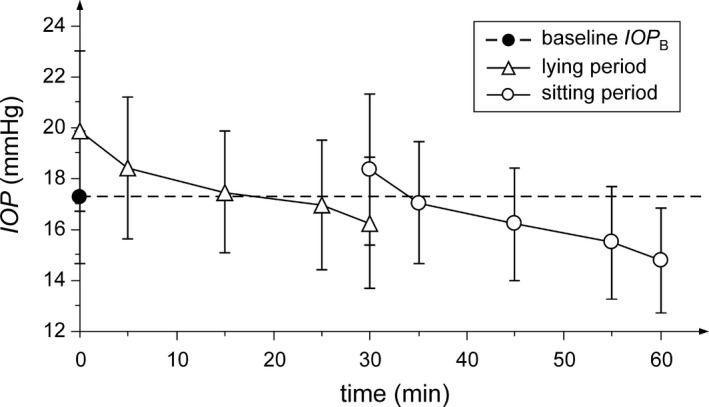
Time course of mean IOP values during the lying (open triangles) and sitting (open circles) period. The graph indicates the IOP increases after each position change and then gradually decreases with time. The half‐sizes of the vertical abscissae correspond to the IOP standard deviations. The black circle and dashed line represent the IOP baseline (in the initial sitting position).

The mean values of IOP during all periods of experiment and standard deviations are presented in Fig. 1. The graph shows baseline IOP in the initial sitting position (17.3 ± 2.6 mmHg), IOP values after lying down and after re‐sitting up; all averaged across subjects. The one‐factor (time) repeated‐measures anova revealed that the values of IOP altered significantly (p* *<* *0.001) with time. The comparison with the baseline using the post hoc Tukey HSD test had shown that the IOP increased significantly immediately after lying down (p* *<* *0.001) with the mean difference 2.6 ± 2.4 mmHg and gradually decreased with time. The IOP in minute 5 was still significantly higher than baseline (p* *=* *0.020) whereas in minutes 15, 25 and 30 it did not differ (p >* *0.99, p >* *0.99, p* *=* *0.070). Immediately after re‐sitting up, the IOP increased again and was significantly higher 1.1 ± 3.3 mmHg than baseline (p* *=* *0.031) and 2.1 ± 3.1 mmHg higher than the last value in the lying position (p* *<* *0.001). The increase was followed by gradual IOP reduction approximately to the baseline in minute 5 after sitting up (p* *>* *0.99) and below the baseline in minutes 15, 25 and 30 after sitting up (p* *=* *0.047, p* *<* *0.001, p* *<* *0.001, respectively). The mean IOP in the final sitting position (60 min from the first measurement) was significantly lower (2.5 ± 1.9 mmHg) than in the initial sitting position. Only 4 subjects (9.1%) had shown higher final IOP compared with the baseline, whereas 29 subjects (65.9%) had shown decrease higher than 2 mmHg. In comparison with the last value in the lying position, the IOP in minutes 5, 15 and 25 after sitting up did not differ significantly (p = 0.36, p > 0.99 and p =* *0.39, respectively), whereas IOP in minute 30 after sitting up was significantly lower 1.5 ± 1.8 mmHg (p <* *0.001).

**Figure 2:**
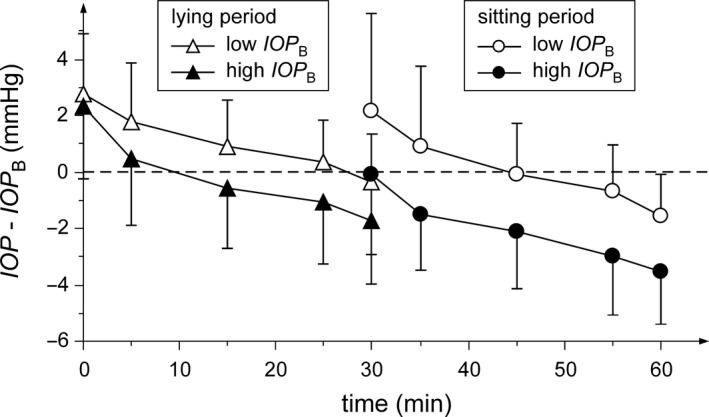
Time course of mean IOP differences from the baseline for the lower (open symbols) and the higher (closed symbols) baseline group. The triangles represent data during the lying period and the circles during the sitting period. The higher baseline group shows higher differences between IOP in both periods than the lower baseline group. The sizes of vertical abscissae correspond to the IOP standard deviations. The dashed line represents zero difference.

We also studied the effects of *IOP*_B_, sex, position and time on IOP changes from *IOP*_B_. The four‐factor repeated‐measures anova proved that the changes significantly differed with position (p <* *0.001), time (p* *<* *0.001) and demonstrated the significant influence of *IOP*_B_ (p* *=* *0.0036) and its interaction with position (p =* *0.042). The IOP was higher in the lying period than in the sitting period with the average difference 1.41 ± 2.4 mmHg. The difference was smaller for the lower baseline (0.9 ± 2.2 mmHg) than the higher baseline (1.9 ± 2.5 mmHg). The influence of the baseline was supported by the significant positive correlation of the baseline and the mean difference between the lying and sitting period (*r *=* *0.332, p =* *0.0277) – the higher baseline leads to the lower IOP in the sitting period. The effect of sex, its interactions with position, time and *IOP*_B_ were insignificant (p = 0.96, p = 0.16, p > 0.99 and p =* *0.59) as well as the interaction of time with *IOP*_B_ (p =* *0.89) and time with position (p =* *0.72). The time courses of the studied IOP changes and their standard deviations for a group of subjects with higher and lower initial IOP are shown in Fig. 2. The average initial IOP for lower and higher baseline IOP groups were 15.2 ± 1.5 mmHg and 19.6 ± 1.2 mmHg. While the IOP of subjects with the lower baseline tend to return approximately to the baseline during the lying period or slightly below at the end of the sitting period, the IOP of those with the higher baseline reached values markedly below the baseline in both periods.

## Discussion

This study performed on a population of healthy volunteers demonstrates that the change between the sitting and supine position significantly affected the IOP. We observed an immediate increase in IOP as a response to both considered changes in the body position and the subsequent gradual decrease with time. The subjects with the higher baseline revealed a lower mean IOP during the final sitting period compared to the lying period. The IOP changes were not influenced by sex. The initial IOP was markedly higher than the final IOP at the end of the experiment, both in the sitting position.

The final IOP after the sitting period declined markedly below the baseline (2.5 ± 1.9 mmHg). A similar effect was also described by Anderson within an unspecified time of calm in the sitting position (Anderson & Grant 1973). We can hypothesize that there is a gradual decrease in IOP on the background of the experiment, which is independent from the position change. Than the resulting IOP could be a composition of this decrease and the position induced changes. Hence, the average difference between the lying and following sitting period reported in our study can be influenced by this effect. The immediate changes after reposition, however, should not be markedly influenced by the gradual decrease. During our experiment, the subjects were calm with open eyes, without accommodation and extensive eye movements; the only activity was the position change after 30 min of lying. The general calming of the entire organism can lead to the decrease of the IOP with time. The higher baseline in IOP showed a higher decline.

The immediate increase (2.6 ± 2.4 mmHg) after lying down compared to the baseline as well as the higher mean IOP in the lying period compared to the sitting period (1.41 ± 2.4 mmHg) are consistent with the published studies in the case of the normal healthy subjects (e.g. Jorge et al. 2010; Fang et al. 2018; Lam et al. 2013; Lee et al. 2012; Lindén et al. 2018; Malihi & Sit 2012; Parsley et al. 1987) as well as the subjects with glaucoma (e.g. Anderson & Grant 1973; Kiuchi et al. 2010; Lindén et al. 2018; Parsley et al. 1987). We found the higher difference between IOP in the lying and the following sitting period in the case of subjects with the higher baseline. It was demonstrated that subjects with glaucoma or eye hypertension (e.g. Parsley et al. 1987; Noël et al. 2001; Hirooka & Shiraga 2003; Katsanos et al. 2017) reached a higher IOP increase when lying down. As these subjects suffer from higher IOP, it is consistent with our findings.

Based on the results of the previous studies (Friberg 1985; Friberg et al. 1987; Arora et al. 2017), the increase of the IOP in the supine position can be caused by the increase of episcleral venous pressure (EVP) after lying down. The EVP, however, reaches the equilibrium gradually with time in contrast to the immediate IOP rise, as discussed by Anderson & Grant (1973). Other possible explanations include the reflux of the aqueous humour from episcleral vessels to the Schlemms’ canal (Friberg et al. 1987) or the passive response of the choroidal circulation to the posture change (Longo et al. 2004).

The gradual IOP decrease in the supine position was not observed previously (e.g. Fang et al. 2018). Due to this decrease, the IOP reached values close to the baseline in minute 15; the higher baseline led to the major decrease. In contrast, Jorge et al. 2010; Lam et al. 2013; Fang et al. 2018 found a significant difference from the baseline after 15 or 30 min in the supine position in the case of normal healthy subjects (Jorge et al. 2010; Lam et al. 2013; Fang et al. 2018). This discrepancy could be explained by the observed dependence of the IOP changes on the baseline (see Fig. 2; subjects with the lower baseline tended to a slower decrease with time) and by the mean initial IOPs in these studies, which were lower compared to our mean baseline. The gradual decrease can be partly connected with the change in pupil size, partly with the above‐mentioned posture‐independent gradual decrease of IOP in time. The pupil is significantly smaller in the supine position compared to the sitting or upright position (Lee et al. 2007) due to parasympathetic nervous system activation in the sitting position (Barrett et al. 2012). The smaller pupil relates to the better outflow of the aqueous humour and IOP reduction.

Recent studies (Lam et al. 2013; Fang et al. 2018) reported a decrease of the IOP after re‐sitting up following the supine position but did not focus on the systematic observation of the IOP dynamics during this period. In our study, we observed increase immediately after re‐sitting up followed by a gradual decrease below the baseline. The sitting up is the reverse situation to the lying down. The EVP should therefore decrease and reach equilibrium over time. Consistently with this, the IOP decreased with time. The immediate increase did not accord, however, with this hypothesis. There must therefore be other effects which strongly influenced IOP simultaneously with the sitting up. When sitting up, the sympathetic nervous system is activated to precede an orthostatic collapse and causes acute increase in the blood pressure and mydriasis (Barrett et al. 2012). This activity can be the cause of the rapid IOP increase. The following decrease can relate to the discussed gradual decrease of IOP on the background of the experiment.

Our results have shown that the IOP significantly changed especially immediately after the position change. This effect should be considered when IOP is measured after the patient's reposition, that is there should be an adequate timing relationship between reposition and measurement. Based on our results, the sufficient time interval must be longer than 5 min. This situation can happen, for example, during 24‐hr monitoring of IOP (for review see Itoop et al. 2016), where the position changes can be a distracting factor. Moreover, if the patients will be calm a longer time before the measurement, the IOP value can be affected by a gradual decrease with time. If the IOP is measured after an extended rest period, there is a risk that the IOP reading will be falsely lower. This effect is stronger for those with higher IOP, that is for glaucoma patients.

The immediate IOP changes, induced by body reposition, were higher than 2 mmHg and from a medical standpoint clinically significant (Qian et al. 2012). Our study included only healthy young subjects. It is known that glaucoma patients are more sensitive to any changes in stress, for example higher fluctuations in IOP during the drinking of water (Salcedo et al. 2018; Susanna et al. 2018) or higher posture‐induced IOP changes (e.g. Tarkkanen & Leikola 1967; Weinreb et al. 1984; Parsley et al. 1987; Noël et al. 2001; Hirooka & Shiraga 2003; Lee et al. 2013; Katsanos et al. 2017). We therefore judge the higher changes in glaucoma patients. These changes could increase the potential risk for people with suspected glaucoma or glaucoma patients.
